# Effectiveness of chiropractic manipulation versus sham manipulation on recurrent headaches in children aged 7–14 years, Protocol for a randomized clinical trial

**DOI:** 10.1186/s12998-019-0262-y

**Published:** 2019-08-23

**Authors:** Susanne Lynge, Jan Hartvigsen, Henrik Wulff Christensen, Werner Vach, Lise Hestbaek

**Affiliations:** 1 Private chiropractic practice, Vivaldisvej 6, 9700 Broenderslev, Denmark; 2 Nordic Institute for Chiropractic and Clinical Biomechanics, Campusvej 55, 5230 Odense M, Denmark; 3 Department Of Sports Science and Clinical Biomechanics, University of Southern Denmark, Campusvej 55, 5230 Odense M, Denmark; 4 Private chiropractic practice, Enghavevej 2, 5800 Nyborg, Denmark; 5 Department of Orthopaedics and Traumatology, University Hospital Basel, Spitalstrasse 21, 4031 Basel, Switzerland

**Keywords:** Chiropractic, Manipulative therapy, Manipulation, Children, Headache, Sham, Recurrent, Placebo

## Abstract

**Background:**

Headache is one of the most common pain symptoms in childhood having a negative impact on many aspects of the lives of affected children, both short-term and long-term. Therefore, it is important to document safe and effective treatment options. Chiropractic spinal manipulation is a commonly used treatment option for these patients, although there are no randomized clinical trials documenting the effectiveness of this in pediatric headache. However, there is moderate evidence for effectiveness of spinal manipulation for adults with tension-type and cervicogenic headaches.

This paper describes the protocol for a two-armed randomized superiority clinical trial aiming to investigate the effectiveness of chiropractic manipulation versus sham manipulation in the treatment of recurrent headache in children aged 7–14.

**Methods:**

Children with weekly headaches for at least six months will be included if they have indications for chiropractic manipulation. The participants will be randomized to either chiropractic manipulation or sham manipulation. Both children and parents will be blinded for allocation. There will be 100 children in each arm and they will answer weekly text messages four weeks prior to treatment and during a four months treatment period. Potential primary outcomes are weekly number of headaches, intensity of headache, medication use and global perceived effect. Secondary outcomes include side-effects and headache status after one year.

An initial outcome data analysis will be performed to inform the choice of primary outcome (adaptive design). Intervention effects will be reported as the difference in mean values between the two treatment arms, Cohen’s effect size and numbers needed to treat.

**Discussion:**

A major strength of this study is its pragmatic nature, where the active treatment group receives chiropractic manipulation according to their individual needs, while both groups continue their use of medication for headache according to their pre-trial habits. Other strengths include an elaborate sham procedure and the weekly outcome reports, reducing recall bias.

If it is possible to develop effective treatment for headache in children, a life course of recurring problems may be altered with potential positive implications for both individuals and society.

**Trial registration:**

ClinicalTrials.gov, identifier NCT02684916.

**Electronic supplementary material:**

The online version of this article (10.1186/s12998-019-0262-y) contains supplementary material, which is available to authorized users.

## Background

Headache is one of the most common pain symptoms in childhood and experienced by up to 80% of 13–15 year old children [1]. Besides the pain and emotional distress, children with recurrent headaches have reduced participation in social activities [2] as well as more school absenteeism and lower academic performance when compared to children without recurrent headache [3]. Socioeconomically disadvantaged children are more prone to headaches [4, 5] and children with headaches generally have poorer overall physical health with higher frequency of asthma, hay fever, anemia, stomach and intestinal illness, obesity and ear infections. Furthermore, they have a higher prevalence of learning disability and attention deficit disorders when compared to children without headaches [5]. Importantly, pediatric headache can be a precursor to potentially severe headache syndromes later in life [6–8].

The etiology of pediatric headache is multifactorial and complex and several causative factors have been proposed, including family history of headache [3], headache medication overuse [9], sleep disturbances [10], depressive/anxious traits [3], stressors [9], a higher rate of divorced parents, fewer peer relations and an unhappy family atmosphere [11]. Headache can also be the result of trauma to the head and neck [12, 13], and in children below the age of six with recurrent and chronic headaches, post traumatic headache is the most frequent type of secondary headache [14]. Headache after minor head, neck or back injury often displays the same symptoms as other types of chronic tension type headache or migraine but they typically have a history of recurrences, and it has been suggested that symptoms may be delayed for months or even years after the injury [15–17]. Finally, prolonged static postures, especially prolonged reading and computeruse, has been associated with headache in 10–13-year-old children [18]. Both trauma and non-appropriate strain of the neck may lead to mechanical problems of the cervical spine. According to the American Migraine Foundation, cervicogenic headache is referred pain perceived in the head from a source in the neck. Cervicogenic headache is a secondary headache, caused by a disorder of the cervical spine and its components: bone, disc and/or soft tissue elements. Numerous pain-sensitive structures exist in the cervical and occipital regions. The junction of the skull and cervical vertebrae have regions that are pain generating, including the lining of the cervical spine, the joints, ligaments, cervical nerve roots and vertebral arteries passing through the cervical vertebral bodies [19]. Therefore, spinal manipulative therapy has been suggested as a treatment option for these headaches [20].Pharmacologic treatment is commonly used in pediatric headaches despite very few randomized controlled trials documenting its effectiveness [7, 21]. Spinal manipulation, spinal mobilization and massage are also used, and in Denmark headache is the second most common complaint among children aged 2 to 17 years old seeking chiropractic treatment [22]. While there are no randomized clinical trials documenting the effectiveness of chiropractic manipulation in pediatric headache [23], there is moderate evidence for effectiveness of spinal manipulation for adults with cervicogenic headache [24] as well as for tension-type headache [25]. Serious adverse events following chiropractic manipulation appear to be very rare [26] and the relative risk of having a minor or moderate adverse event after high velocity thrust spinal manipulation was significantly less than after taking medication which is often prescribed for painful conditions [27]. In a review of compensation claims following chiropractic treatment in Denmark and Norway between 2004 and 2012, there were no reports of harm or injury resulting in compensation claims in children age 0–18 years old [28].

In this paper, we describe the protocol for a two-armed randomized superiority clinical trial aiming to investigate the effectiveness of chiropractic manipulation versus sham manipulation in the treatment of recurrent headache in children aged 7–14. Both groups receive oral and written lifestyle advice that is beneficial to headache patients, and they can use medication according to individual needs throughout the trial. Consequently, our design allows us to investigate the specific effect of chiropractic manipulation on top of standard care.

## Method

### Procedures

#### Recruitment

The recruitment for this trial began November 2015, by January 2nd 2019, 180 children were included and recruitment is expected to be concluded by June 2019. School principals in the region of Northern Jutland, Denmark, are contacted by telephone and asked for permission to distribute an invitation (Additional file 1) to all 1st -7th grade children through the Danish School Information Network, which is the official communication line between the school, the child and the parents. Furthermore, information is distributed through local newspapers, television and radio, as well as social media. Parents with children aged 7–14 suffering from recurrent headaches are invited to contact the project clinic for screening for inclusion in the trial.

#### Setting and pre-randomization procedure

The project clinic is a primary care chiropractic practice owned by the primary investigator. Upon contact, thorough information about the study is given by a project secretary on the telephone, including information about the two treatment arms and blinding procedures. If the parents decide to participate, the secretary will check inclusion/exclusion criteria (see Table 1) and send them written information as well as an informed consent form, including a pre-stamped return envelope. Within a week, the consent form, signed by a parent/guardian, should be sent to the Nordic Institute of Chiropractic and Clinical Biomechanics (NIKKB), where a research assistant and data manager will contact the parents by telephone and initiate a four-week pre-treatment observation period. During this period, the parents and the child together answer the following three questions every Sunday, sent to the parents as a text message (SMS) on their cell phone:

1. “How many days has <child’s name> had a headache this week? Choose a number between 0 and 7.”
2. “How will you rate the pain on a scale from 0-10, where 0 is no pain and 10 is the worst pain you can imagine?”
3. “How many pills for headache has < child’s name> taken this week? 0: none, 1: 1-4, 2: more than 4 pills.”

**Table 1:** Inclusion and exclusion criteria with their respective time points

Inclusion criteria	Time point for checking*
7–14 years old	1
at least one episode of headache per week for at least half a year prior to the pre-treatment period	2
At least one musculoskeletal dysfunction identified by the primary investigator as a movement restriction in one or more of the joints suitable for chiropractic manipulation treatment at the time of the initial screening.	3
written informed consent from the holder(s) of custody	1
a mobile telephone in the family and ability to report weekly headache data	1
ability to read and speak Danish at an adequate level to comply with study requirements	2
*Exclusion criteria*	
contraindications for joint manipulation (Additional file 3)	3
receiving any treatment by a health care professional for headache within the last three months. The only exception being prescription pain medication	2
failure to report baseline data during four weeks prior to randomization	2

*Time points for checking inclusion and exclusion criteria: 1 = prior to start of four weeks pretreatment period; 2 = prior to screening visit; 3 = at screening visit

Before the start of the SMS-track, parents are contacted by telephone by a research assistant at NIKKB, instructing them to report the average of severity of all headaches during the previous week to the second question. Parents answer using the reply function, and the answers are automatically registered and stored in a database. If they do not reply, they will automatically get a SMS reminder the following Tuesday. The SMS-system is an efficient way to obtain frequent information and has been proven feasible, also in this age group [29]. Upon initiating this procedure, the data manager at NIKKB also sends an email to the parents containing a link to an electronic questionnaire through Survey Exact (https://www.surveyxact.com/) inquiring about the characteristics of the child’s headache problem, lifestyle, previous trauma, previous treatment, family history of headache and general health (Additional file 2). After the four-week pre-treatment observation period, the child and parents are scheduled for a screening at the chiropractor’s office after checking further inclusion/exclusion criteria (see Table 1).

#### Clinical baseline examination

During the initial clinical examination (Additional file 4) visit, the chiropractor will perform a thorough neurologic examination to identify findings requiring immediate medical attention or referral to a pediatrician connected to the project (Additional file 5). If none are found, the chiropractor examines the spine, pelvis and temporomandibular joints for biomechanical dysfunctions to assess whether the child is a candidate for chiropractic manipulation (Additional file 6) and if there are no exclusion criteria present, the child will be offered to enter the trial (see Table 1).

At this visit, all participants and their parents are informed about possible side effects known to chiropractic treatment and are told to keep track of all such effects in order to be able to report them to the chiropractor. They are also told that information about trauma experienced during the trial period will be noted at each visit as well. If the participant and his/her parents then consent to participate in the trial, the participant is randomized.

#### Randomization

Participating children are randomized with 1:1 allocation using random block size with the software nQuery Advisor [30] by the data manager at NIKKB upon receipt of the consent form. Group assignment is noted in opaque envelopes and sent to the project clinic.

When the child has completed the 4 weeks pre-treatment period and is found eligible for inclusion at the clinical examination visit, the randomization envelope from NIKKB will be opened by the primary investigator and the trial period of 4 months will begin. Neither the child nor the parents are informed about group allocation.

### Interventions

All participants from both groups receive oral and written advice about lifestyle beneficial to headache patients in general. Healthy habits that include regular sleep, exercise, fluids and meals are considered a cornerstone of treatment [21]. Therefore, we have designed a list of basic advices concerning lifestyle that we think are commonly known and generally accepted in the population. We have made these advices as simple and understandable as possible in order to make them easily applicable to the participants (Additional file 7).

The parents are then asked to leave the room, and participants in both groups are examined via static and motion palpation of the entire spine, pelvis and temporomandibular joints. After this examination, either the chiropractic manipulation or the sham manipulation is administered. Thus, each consultation resembles a normal consultation with a chiropractor in both groups.

#### Chiropractic manipulation

The children in the chiropractic manipulation treatment group receive chiropractic manipulation as indicated according to the examination utilizing a specific contact, high-velocity, low- amplitude, short lever technique with a thrust given according to the determined line of drive, resulting in an audible joint cavitation [31]. If treatment is indicated in the lumbar spine or the pelvis, the child will lie on the side as indicated by the clinical findings, with the upper knee bent and the chiropractor supporting the upper shoulder while stabilizing the patient’s leg and hip with her own leg and delivering a high-velocity, low-amplitude thrust. If manipulation of the thoracic spine is determined to be indicated, a high velocity, low-amplitude thrust will be given with the patient in a prone position. If treatment in the neck is indicated, the child will sit on a chair and the chiropractor will stand behind the patient while contacting the spinous process of the joint to be manipulated and a high velocity, low-amplitude thrust is given. All treatments are modified to fit the age and size of the child as well as individual joint characteristics.

To reflect daily clinical practice, this treatment is given pragmatically, i.e. the number and frequency of treatments, as well as the joints treated, are based on the chiropractor’s individual evaluation at each visit. Number and dates of treatment as well as joints treated will be noted.

#### Sham manipulation

The placebo treatment is very similar to the placebo treatment described by Chaibi et al. [32] utilizing the same treatment positions and patient placements as used in the chiropractic manipulation group. The sham manipulation consists of a broad, non-specific contact, low-velocity, low-amplitude gentle push in a non-intentional direction. All contacts are performed away from the spinal column without soft tissue pretension, so no joint cavitation occurs. The participant will first lie on the right side with the left knee bent where the chiropractor holds on to the participant’s left shoulder and the left gluteal region while delivering the light non-intentional push, equivalent to approximately half a pound of pressure, on the lower, lateral part of the gluteal muscle with no cavitation from the lumbar or sacroiliac joints. Afterwards the child will lie prone on the treatment table while the chiropractor places a hand on each of the participant’s lateral scapular edge and delivers a light push, equivalent to approximately half a pound of pressure, with no resulting joint cavitation. Finally the participant sits in a chair and the chiropractor puts a hand on the participant’s left trapezius muscle, puts a de-activated activator instrument [33] on the chiropractors own left forearm and ask the participant to turn the head to the right. At the moment when the participant turns the head, the chiropractor will give a click with the de-activated activator. This procedure will then be repeated on the other side, again with no cavitation of any joints. Each participant in this group is given a total of eight visits with sham manipulation with four visits during the first month of participation, two the second, one the third and one during the fourth attempting to resemble a common course of care in a chiropractic practice.

#### Blinding

Blinding of the participating chiropractor in this trial is obviously impossible. Blinding of the participants is attempted by not revealing the group allocation and by including a sham manipulation that closely resembles the active treatment. Furthermore, eight visits are given in the sham manipulation group to resemble the estimated number of treatments in the chiropractic manipulation treatment group to reduce attention bias. Also, in the control group an activator treatment [33] used on the chiropractor’s own arm in order to produce a noticeable clicking noise resembling a joint cavitation. Blinding of the parents is attempted by not inviting them to observe the treatment. At the end of the treatment period, participating children and their parents receive a question asking them, which of the two groups they believe they have been participating in.

At all sessions in both groups, parents are allowed to be present during the interview where the chiropractor will ask about headaches, side effects experienced after the previous treatment according to a checklist (Additional file 8), and the participant will also be asked if he/she has experienced any trauma since the last visit, and if so, the characteristics of the trauma.

### Collection of data

#### Baseline data

Baseline data will be collected prior to the pre-treatment period by a questionnaire including the following items:

- age
- sex
- duration and frequency of headache problem prior to inclusion
- characteristics of headache problem
- prior examinations and/or treatment of headache
- family history of headache
- trauma to head and/or neck prior to inclusion
- neck and/or back pain
- other health problems
- diet, sleep and exercise habits
- sports
- socioeconomic status
- smoking in the home

#### Outcome data

Throughout the study period, parents and participants answer the same weekly text messages on their mobile telephone as during the pre-treatment observation period, independent of the chiropractor.

At the end of the 4 months of treatment, all participating families receive a final text message including three questions:

1. “How satisfied is <child’s name> with participation in this trial on a scale from 0-10, where 0 is the worst and 10 is the best you can imagine?”
2. “How has the headache changed since <child’s name> started the treatment at the chiropractor? 1. almost gone/disappeared. 2. much better. 3. a little better. 4. same. 5. a little worse. 6. much worse. 7. worse than ever.”
3. “In this trial there have been two groups. Do you think that <child’s name< was in group 1, who had standard chiropractic treatment or in group 2, that DID NOT have standard chiropractic treatment (please answer 1 or 2)?”

All participating families receive a questionnaire 1 year after entering the trial, including questions about headache status of the child as well as other treatments received for headache (Additional file 9).

#### Additional information collected during the trial period by the chiropractor

- number of treatments
- articulations treated (recorded at each visit)
- numbers, severity and character of traumas/accidents experienced between visits while participating in the trial (recorded at each visit)
- side effects experienced between two treatments (type, timing and duration; recorded at each visit)
- other diseases reported/identified during treatment period
- number and reasons for drop-out

### Variables

#### Primary outcomes

*For each week we will consider three variables based on the weekly SMS:*

1. Number of days with headache per week
2. Headache intensity on a pain scale from 0 to 10 per week
3. Number of headache pills per week

To catch the effect of the intervention, we will consider the average values during the pre-treatment period and the final 4 weeks of the study period (week 14–17). Three primary outcomes are then given by the change over time, i.e. the difference between the average values from the final 4 weeks and from the pre-treatment period. The primary outcomes are prioritized as listed above. A fourth primary outcome is the global perceived effect (GPE) after 4 months based on the final SMS.

#### Secondary outcomes

- Character and duration of side effects experienced after treatment
- Headache status after 1 year (questionnaire)
- General perceived effect after 8 months for children reporting little effect, no effect or worsening of headache in the final SMS after the treatment period and subsequently received care from the pediatrician if they had received chiropractic manipulation, or a course of chiropractic manipulation if they had received sham manipulation

### Follow-up procedures after the treatment period

Children in the sham manipulation group reporting little effect, no effect or worsening of headache in the final SMS after the treatment period are offered free chiropractic care, similar to the care delivered in the manipulation group, during the trial for 4 months following the trial period. After the four-month post-trial treatment period, parents will receive a final text message, identical to the one they received after participating in the trial regarding the effect of the treatment. If they still report little effect, no effect or worsening after treatment, they will be offered a consultation with the trial pediatrician.

Children in the chiropractic treatment group reporting little, no effect or worsening after treatment will be offered a consultation with the pediatrician, and after 4 months they will receive a final text message, identical to the one they received after participating in the trial.

### Sample size considerations

Sample size depends on the expected change on the primary outcome as well as the average values of the outcome and the inter-individual variation. Because this is the first study using an assessment of these endpoints shared by parents and children in a child population aged from 7 to 14 years, we do not have any reliable information on the population means and variations in the intended primary outcomes. We hence decided to perform a sample size calculation when the data collection was completed for 50 children in each treatment group. We decided to use number of weekly headache days as basis for this calculation, since this is our initially highest prioritized primary outcome candidate. The mean number of weekly headache days during the 4 weeks pre-treatment was 11 days (2.75 days/week). The standard deviation of the change in number of weekly headache days from pre- to post treatment was 5.58 and normally distributed. Based on consensus among the authors, 20% of weekly headache days was decided to be a clinically relevant reduction, because it is close to half a day per week in relation to the mean pre-treatment weekly headache days. Thus, a sample size of 100 children in each group is needed to detect a difference of 20% of the pretreatment mean (0.55 day/week) with a power of 80% and a significance level of 5%. Calculations were performed in nQuery Advisor [30]. Allowing for a 20% drop-out rate, the aim for inclusion will be 240 children.

### Statistical analyses

As mentioned above, our interest is in four potential endpoints, which we have prioritized according to clinical relevance. However, due to lack of experience with using these outcomes in this setting, we do not know whether they are measured in a reliable manner and whether they show a population variation which makes them suitable as an outcome in an RCT. For example, we cannot exclude that there is little variation in some of the intended outcomes across children or that we observe associations with baseline variables which are lower than expected and/or difficult to explain. Such insights may lead to a change in the prioritization of the outcome variables.

We will hence conduct an initial outcome data analysis. With respect to the three SMS questions we will inspect the individual trajectories regarding smoothness and visibility of improvements or deteriorations at the individual level. Next, we will relate the 4 weeks pre-intervention averages and standard deviations to all baseline characteristics and to each other in order to check whether the observed association patterns can be explained in a reasonable manner. With respect to the change from pre-intervention (four-week averages prior to intervention) to post-intervention (four-week averages at end of trial period) we will consider the joint distribution to judge the existence and magnitude of floor and ceiling effects and to inform the choice between absolute or relative changes. We will also depict the distribution of the change variables themselves, in particular with respect to the degree of variation. Further, we will compare the distribution of the three change variables to judge both the general association as well as specific combination patterns, for example the occurrence of a clear improvement in one question but a stagnation in another question. These analyses will also be stratified by age and gender to judge whether such characteristics may influence the degree and pattern of association. If no clear patterns emerge, the investigation will be extended to a multivariable analysis of all pre- and post-intervention measurements of the three questions. GPE will be included in the analysis of the change variables.

The initial outcome data analysis will be performed by an independent statistician, who will obtain a copy of the data set with baseline characteristics, the weekly results for the three SMS questions and the GPE of the first 100 children. No information on treatment group membership will be provided. The statistician will prepare a statistical report to be distributed among the research team. The research team and the statistician will discuss the results and prepare a further short report summarizing the insights gained and potentially deciding on a reprioritization. We do not use a priori defined criteria for the decision to change the prioritization. In general, many different properties may pop up which may influence the decision. Consequently, it is hard to pre-specify an algorithm. Initial outcome analyses have rarely been performed until now, so there is no established framework for this. However, the decision will be based on a combination of the statistical findings and theory. We will therefore report the entire process for clarity (statistical report as well as a transparent report of the decision process). All analyses will be blinded and we can therefore exclude any undue influence on the final results of the study. Both reports will be finished prior to randomizing the last child in the study and will be included in the supplementary material of the first research publication.

In the primary analyses, we will visualize and describe the distribution of the four primary outcomes in each treatment arm. Intervention effects will be assessed by considering the difference in mean values between the two treatment arms, reported together with 95% confidence intervals and *p*-values. In addition, Cohen’s effect size defined as the difference in mean values divided by the common standard deviation will be reported.

Multiplicity due to using four primary outcomes will be taken into account by applying the fixed sequence test principle [34] using the prioritization determined in the initial outcome analysis as the a-priori order of hypotheses. For all four primary outcomes, inference will be based on a least squares regression model. For each change score the baseline level will enter as covariate, and for the global perceived effect the number of days with headache per week will enter the model. In case the initial outcome analysis points to correlations between patient characteristics and outcomes above 0.3, these patient characteristics will be added as additional covariates. A responder analysis will be performed, reporting the proportion in each group reporting 20, 25, 50 and 75% improvement compared to baseline for the three change scores. Furthermore, numbers needed to treat, with success defined as 20% improvement, will be estimated.

Secondary outcomes will be analyzed similarly.

Patient and treatment characteristics as well as drop out frequencies and patterns will be described within each treatment group. Single missing values in the SMS data will be ignored, as long as there is at least one value available within the last 4-weeks periods. Patients with missing answers to all 4 weeks in last period will be ignored (complete case analysis). Missing values in baseline characteristic will be replaced by regression-based predictions from the other baseline variables, using least squares regression to predict continuous variables, and ordinary, ordinal or multivariate logistic regression for binary, ordinal and categorical variables.

Multiple imputation will be used to conduct sensitivity analyses with respect to handling missing values. These will be based on a chained equation approach (as implemented in Stata’s mi command) considering simultaneously all outcomes and baseline variables. Analyses of data will be conducted by a researcher, otherwise not involved in the project and who will be blind for the group coding. The clinical investigators will not be involved in the analyses.

## Discussion

Chronic or recurrent headache in children can have a negative impact on their participation, academic performance, physical health etc. [2, 3, 5]. Considering the potential long-term consequences of this, as well as the likelihood of pediatric headaches to continue into adulthood [6–8], it is important to develop safe and effective evidence-based treatment options. To our knowledge, this is the first randomized controlled trial investigating the effect of chiropractic manipulation on children with recurrent headaches. The results of the trial will inform future research in this area as well as clinical practice relating to non-pharmacological treatment of recurrent headache in children aged 7 to 14.

An adaptive design was chosen in relation to prioritizing the primary outcomes, as we cannot build on experience with these outcomes in this setting. Adaptive designs can be applied across all phases of clinical research and are more flexible by using results accumulated during the trial to modify the trial’s design or course in accordance with pre-specified rules [35]. Interim non-comparative analyses can be used to inform sample-size considerations as well as choice of outcome measure [36], which is particularly useful in areas where there are no established traditions or recommended core sets of outcomes, as is the case in this study. This design allows for correction of potential misjudgments in the priorities of outcomes [37], and since the interim analyses are pre-planned and performed by an independent statistician, the scientific standard is not compromised.

The involvement of only one practitioner performing all treatments ensures a high level of homogeneity in the treatments. However, it can also represent a weakness because the results of the treatment depend on the ability of only one practitioner. This weakness is accentuated by the poor reliability reported for palpatory findings [38] which are used to identify the biomechanical dysfunctions suitable for manipulative treatment.

The parent-child interaction may bias the answers of both child and parent [21] because there can be a discrepancy between the child and the parent’s report of the child’s headache [39]. Therefore, it is important that the same person reports on the text message headache diary every week during the entire trial and this represents a challenge for some children living in divorce families with inadequate communication between the parents. This is especially true for the SMS-question regarding use of medication because the children may not always be aware of what type of medication they receive and for what. Reporting of medication used can also depend on the age of the child. Some children are allowed to carry their own non-prescription medication for headache and use it according to own needs without asking the parents first, and therefore memory bias can be an issue in this age group.

A major strength of this study is its pragmatic nature, performed in a normal clinical setting, where the chiropractic manipulation treatment group receives treatment according to their individual needs in terms of areas in the spine being treated and the number of treatments given. In addition, parents and children in both groups can continue their usual self-care for headache if and when they want to. First of all, this allows us to investigate the specific effect of chiropractic manipulation and secondly, is a requirement for ethical reasons because we investigate children who suffer from recurrent and in some cases severe headache and/or migraine. Further strengths include the elaborate sham procedures and the SMS registration of outcomes, reducing recall bias. Finally, it should be noted that both the chiropractic manipulation group and the sham manipulation group are attended to by the same chiropractor at every visit, reducing the risk of equipoise bias in the treatment group, and ensuring that the exact same procedure is performed on all participants (except for manipulation/sham manipulation).

If it is possible to develop effective treatment for headache in children, a life course of recurring problems may be altered with potential positive implications for both individuals and society. And because there are no reports of serious side effects to this type of manipulative therapy in children in the scientific literature, besides temporary reddening and soreness, the possible implications in terms of improved health and wellbeing could be considerable.

## Additional files

Additional file 1: Written invitation to parents (DOCX 13 kb)

Additional file 2: Baseline questionnaire on headache and lifestyle (DOCX 19 kb)

Additional file 3: Considerations and contraindications to chiropractic treatment (DOCX 13 kb)

Additional file 4: a: Screening A. b: Screening B. (DOCX 24 kb)

Additional file 5: Immediate referral to pediatrician (DOCX 13 kb)

Additional file 6: Chiropractic examination (DOCX 12 kb)

Additional file 7: Advices given to all participants before randomization (DOCX 13 kb)

Additional file 8: Data collection at each visit on side effects and trauma (DOCX 14 kb)

Additional file 9: 1 year follow up questionnaire on headache (DOCX 29 kb)

## Abbreviations

GPE: General perceived effect
NIKKB: Nordic Institute of Chiropractic and Clinical Biomechanics
RCT: Randomized controlled trial
SMS: Short Message System
