# Supplementary material for: Effectiveness of chiropractic manipulation versus sham manipulation on recurrent headaches in children aged 7–14 years, Protocol for a randomized clinical trial
Source: Chiropr Man Therap. 2019 Aug 23;27:40. doi: 10.1186/s12998-019-0262-y (PMC6706934; doi:10.1186/s12998-019-0262-y)
Supplement: Supplementary file 1 — Written invitation to parents (DOCX 13 kb) [file 12998_2019_262_MOESM1_ESM.docx]

Appendix 1

**Invitation to parents**

Clinical trial investigating chiropractic treatment for children with chronic headache

Is your child between 7-14 years old?

Has he/she suffered from recurrent headache for at least half a year?

Does he/she have a headache at least once a week?

This is an invitation for your child to participate in a clinical trial investigating the effect of chiropractic spinal manipulation on children, who suffer from headache. The trial is done in collaboration between chiropractor Susanne Lynge Rosing, pediatrician Olav Bennedbæk and Nordic Institute of Chiropractic and Clinical Biomechanics (NIKKB) and University of Southern Denmark (SDU).

Your child will attend a physical examination conducted by chiropractor Susanne Lynge Rosing. If this examination indicates that your child has a health problem requiring immediate medical attention, he/she will be referred directly to pediatrician Olav Bennedbæk. If not, he/she will receive a thorough chiropractic examination of the entire spine. If this examination indicates that your child has any spinal dysfunction suitable for chiropractic spinal manipulation he/she will be offered to enter the trial.

Your child will be able to participate for four months and after that the result is evaluated. If your child does not obtain a beneficial result, he/she will be offered additional examination and treatment either by pediatrician Olav Bennedbæk or chiropractor Susanne Lynge Rosing. To be able to participate both parents must sign a written informed consent formular.

You can choose whether your child should participate at Børneklinikken, Gugvej 185, 9210 Aalborg S.Ø. or at Kiropraktisk Center Brønderslev, Vivaldisvej 6, 9700 Brønderslev.

Participation in the trial is free

If Your child wants to participate, please contact:

Project Manager Susanne Lynge Rosing, phone: 53822544

email: sl@kiropraktiskcenter.dk
