# Supplementary material for: Effectiveness of chiropractic manipulation versus sham manipulation on recurrent headaches in children aged 7–14 years, Protocol for a randomized clinical trial
Source: Chiropr Man Therap. 2019 Aug 23;27:40. doi: 10.1186/s12998-019-0262-y (PMC6706934; doi:10.1186/s12998-019-0262-y)
Supplement: Supplementary file 2 — Baseline questionnaire on headache and lifestyle (DOCX 19 kb) [file 12998_2019_262_MOESM2_ESM.docx]

| **Headache in children - Baseline questionnaire**  If there are questions you find difficult to answer, we would like you to choose the most appropriate answer. |
| --- |

Age

(1) ❑ 7 years

(2) ❑ 8 years

(3) ❑ 9 years

(4) ❑ 10 years

(5) ❑ 11 years

(6) ❑ 12 years

(7) ❑ 13 years

(8) ❑ 14 years

Gender

(1) ❑ Boy

(2) ❑ Girl

How long have you suffered from headache?

(2) ❑ ½ - 1 year

(4) ❑ 1 - 3 years

(3) ❑ More than 3 years

How often do you have a headache?

(1) ❑ 1 - 2 days/week

(2) ❑ 3 - 5 days/week

(3) ❑ Almost every day

How long does your headache last?

(1) ❑ Less than 2 hours

(2) ❑ From 2hours to half a day

(3) ❑ All day

(4) ❑ All day and night

When does your headache typically begin?

(1) ❑ Morning

(5) ❑ Before noon

(2) ❑ Afternoon

(3) ❑ Evening/night

(4) ❑ At different times

Where is your headache most commonly located?

(1) ❑ All over the head

(2) ❑ Backside of the head

(3) ❑ One or both sides of the head

(4) ❑ Forehead

(5) ❑ Behind one eye

(6) ❑ Different locations

(7) ❑ It varies a lot

Do you have other symptoms with your headache?

|  | Yes | No |
| --- | --- | --- |
| Nausea | (2) ❑ | (1) ❑ |
| Vomiting | (2) ❑ | (1) ❑ |
| Dizziness | (2) ❑ | (1) ❑ |
| Stomach pain | (2) ❑ | (1) ❑ |
| Visual disturbances | (2) ❑ | (1) ❑ |
| Spots in front of eyes | (2) ❑ | (1) ❑ |
| Tingling in arms | (2) ❑ | (1) ❑ |
| Sensitive to light | (2) ❑ | (1) ❑ |
| Sensitive to sound | (2) ❑ | (1) ❑ |
| Others | (2) ❑ | (1) ❑ |

Does any of these give you a headache?

|  | Yes | No |
| --- | --- | --- |
| Neck pain | (2) ❑ | (1) ❑ |
| Back pain | (2) ❑ | (1) ❑ |
| Stress | (2) ❑ | (1) ❑ |
| Sitting down for long periods | (2) ❑ | (1) ❑ |
| Reading | (2) ❑ | (1) ❑ |
| Sport | (2) ❑ | (1) ❑ |
| Computer/TV | (2) ❑ | (1) ❑ |
| Menstrual period | (2) ❑ | (1) ❑ |

Does any of these relieve your headache?

|  | Yes | No |
| --- | --- | --- |
| Lying down | (2) ❑ | (1) ❑ |
| Sleep | (2) ❑ | (1) ❑ |
| Eat something | (2) ❑ | (1) ❑ |
| Drink something | (2) ❑ | (1) ❑ |
| Go outside for fresh air | (2) ❑ | (1) ❑ |
| Sport | (2) ❑ | (1) ❑ |
| Medication | (2) ❑ | (1) ❑ |

Have you had any of these within the last year?

(2) ❑ Neck pain

(1) ❑ Back pain

Do you wear braces on your teeth?

(2) ❑ No

(1) ❑ Yes

How often do you take non-prescription medication for headache?

(5) ❑ Never

(2) ❑ 1 – 3 times/month

(3) ❑ 1 – 3 times/week

(4) ❑ More than 3 times/week

Do you take prescription medication for your headache?

(1) ❑ No

(2) ❑ Yes

What is the name of your prescription medication for headache?

________________________________________
________________________________________
________________________________________
________________________________________
________________________________________
________________________________________

How often do you take it?

(2) ❑ 1 – 3 times/month

(3) ❑ 1 – 3 times/week

(4) ❑ More than 3 times/week

Do you take medication regularly for other conditions?

(2) ❑ Yes (If yes, for what diseases?): _____________________

(1) ❑ No

Did you get any of these examinations due to your headache?

|  | Yes | No |
| --- | --- | --- |
| Examination by family doctor | (2) ❑ | (1) ❑ |
| X-rays of your head or neck | (2) ❑ | (1) ❑ |
| MR/CT scan of your head or neck | (2) ❑ | (1) ❑ |
| Blood test | (2) ❑ | (1) ❑ |
| Examination by pediatrician | (2) ❑ | (1) ❑ |

Choose the number below that best describe your typical headache:

(1) ❑ 0=no pain

(2) ❑ 1

(3) ❑ 2

(4) ❑ 3

(5) ❑ 4

(6) ❑ 5

(7) ❑ 6

(8) ❑ 7

(9) ❑ 8

(10) ❑ 9

(11) ❑ 10=worst pain ever, stops your activity

Have you received any treatment for your headache?

(2) ❑ Yes

(1) ❑ No

Who has treated you?

|  | Yes | No |
| --- | --- | --- |
| Family doctor | (2) ❑ | (1) ❑ |
| Pediatrician | (2) ❑ | (1) ❑ |
| Physiotherapist | (2) ❑ | (1) ❑ |
| Chiropractor | (2) ❑ | (1) ❑ |
| Massage therapist | (2) ❑ | (1) ❑ |
| Reflexologist | (2) ❑ | (1) ❑ |
| Other | (2) ❑ | (1) ❑ |

When was your last treatment for headache?

(1) ❑ Within the last ½ year

(2) ❑ More than ½ year ago

How many times have you hurt your head and/or neck without seeking a doctor or emergency room?

(1) ❑ 0 times

(2) ❑ 1 – 3 times

(3) ❑ More than 3 times

How many times have you hurt your head and/or neck, causing you to contact family doctor or emergency room?

(1) ❑ 0 times

(2) ❑ 1 – 3 times

(3) ❑ More than 3 times

Have you been hospitalized because of an accident to your neck and/or head?

(2) ❑ Yes

(1) ❑ No

Have you ever hurt you head and/or neck in any of these?

|  | Yes | No |
| --- | --- | --- |
| Car accident | (1) ❑ | (2) ❑ |
| Fall of bike | (1) ❑ | (2) ❑ |
| Fall from more than 2 meters distance | (1) ❑ | (2) ❑ |
| Fall off/on trampoline | (1) ❑ | (2) ❑ |
| Fall off horse | (1) ❑ | (2) ❑ |
| Hit by another player in any contact sport | (1) ❑ | (2) ❑ |
| Experienced episode of violence to you | (1) ❑ | (2) ❑ |

Have you had any days off from school due to trauma?

(3) ❑ No

(2) ❑ Yes, once

(1) ❑ Yes, more than once

Have you ever had a concussion?

(1) ❑ Yes

(2) ❑ No

How many days off from school do you have on average?

(1) ❑ Less than 5 days/year

(2) ❑ 5 - 20 days/year

(3) ❑ More than 20 days/year

What are the most common reasons for your days off from school?

|  | Yes | No |
| --- | --- | --- |
| Headache | (1) ❑ | (2) ❑ |
| Neck pain | (1) ❑ | (2) ❑ |
| Back pain | (1) ❑ | (2) ❑ |
| Common cold | (1) ❑ | (2) ❑ |
| Earache | (1) ❑ | (2) ❑ |
| Menstruation | (1) ❑ | (2) ❑ |
| Stomach pain | (1) ❑ | (2) ❑ |
| Don’t want to go school | (1) ❑ | (2) ❑ |
| Other reasons | (1) ❑ | (2) ❑ |

Other possible reasons for days off from school:

________________________________________
________________________________________
________________________________________
________________________________________
________________________________________
________________________________________

How many days off sick have you had the last year due to headache

(1) ❑ 0 days

(2) ❑ 1 – 5 days

(3) ❑ 5 – 20 days

(4) ❑ More than 20 days

Does anyone in your family have headaches?

|  | Yes | No |
| --- | --- | --- |
| Mother | (1) ❑ | (2) ❑ |
| Father | (1) ❑ | (2) ❑ |
| Siblings | (1) ❑ | (2) ❑ |

Are you allergic to any of these (you can mark more than one)?

|  | Yes | No |
| --- | --- | --- |
| Pollen | (2) ❑ | (1) ❑ |
| Certain foods | (2) ❑ | (1) ❑ |
| Perfume/soap | (2) ❑ | (1) ❑ |
| Food additives | (2) ❑ | (1) ❑ |
| Certain animals | (2) ❑ | (1) ❑ |
| Dust mites | (2) ❑ | (1) ❑ |
| Smoke | (2) ❑ | (1) ❑ |
| Others | (2) ❑ | (1) ❑ |

Do you have stomach pain?

(4) ❑ Often (more than 1/month)

(3) ❑ Sometimes (6-12/year)

(2) ❑ Rarely (1-5/year)

(1) ❑ No

Does anybody smoke in your home?

(2) ❑ Yes

(1) ❑ No

How do you get to school in the morning?

(1) ❑ Bike

(2) ❑ Walk

(3) ❑ Bus

(4) ❑ By car

(5) ❑ Other

How many hours per week do you do sports?

(1) ❑ 0

(2) ❑ 1 – 3/week

(3) ❑ More than 3/week

What type of sports do you do?

|  | Yes | No |
| --- | --- | --- |
| Ball game | (2) ❑ | (1) ❑ |
| Running | (2) ❑ | (1) ❑ |
| Athletics | (2) ❑ | (1) ❑ |
| Riding | (2) ❑ | (1) ❑ |
| Fitness | (2) ❑ | (1) ❑ |
| Biking | (2) ❑ | (1) ❑ |
| (other) | (2) ❑ | (1) ❑ |

How many hours per day do you spend on computer/TV/iPad/mobile phone?

(1) ❑ 0 - 1 hour/day

(2) ❑ 2 - 4 hours/day

(3) ❑ 5 - 6 hours/day

(4) ❑ More than 6 hours/day

How many hours do you sleep per 24 hours?

(1) ❑ 6 - 8

(2) ❑ 9 - 10

(3) ❑ 11 - 12

Do you sleep well?

(2) ❑ Yes

(1) ❑ No

For girls: Do you have menstruation?

(2) ❑ Yes (at what age did it start? ❑ 8-11 ❑ 12-14)

(3) ❑ No

For girls: Do you take birth control pills?

(2) ❑ No

(1) ❑ Yes

Do you use glasses/contact lenses?

(2) ❑ No

(1) ❑ Yes

| Thank you very much for your answers |
| --- |
