# Supplementary material for: Effectiveness of chiropractic manipulation versus sham manipulation on recurrent headaches in children aged 7–14 years, Protocol for a randomized clinical trial
Source: Chiropr Man Therap. 2019 Aug 23;27:40. doi: 10.1186/s12998-019-0262-y (PMC6706934; doi:10.1186/s12998-019-0262-y)
Supplement: Supplementary file 3 — Considerations and contraindications to chiropractic treatment (DOCX 13 kb) [file 12998_2019_262_MOESM3_ESM.docx]

Appendix 3

**Possible contraindications to chiropractic spinal manipulation**

Extra precaution and consideration should be taken if:

1. Visual disturbances, pupillary dysfunction or papilledema
2. Difficulty breathing
3. High blood pressure
4. Fever
5. Difficulty urinating or passing stools
6. Stomach pain with continuous vomiting, jaundice, chronic diarrhea
7. Rapid weight fluctuation
8. Unexplainable bruising, edema, swelling, psoriasis
9. Swollen lymph glands in the throat/neck, enlargement of thyroid, hyperhidrosis, polyphagia, temperature intolerance
10. Swollen and painful joints, symptoms of arthritis, pain/swelling of bones and/or muscles, pain/unable to stand on leg(s), pain at night. Visible signs of skeletal malformation/other congenital malformations. Acute damage to joints/sprains. Also attention on possible violence/abuse.
11. Suspicion of fracture(s)
12. Loss of consciousness, black outs, paresthesia, cramps, paralysis, abnormal movement or speech, kinesthesia, changes in personality, depression, hallucination.

add.10: If congenital malformation or fracture is suspected, an x-ray examination can be performed.

References:

Anrig and Plaugher: Pediatric Chiropractic, Second Edition, 2013, Lippincott Williams & Wilkins, p.690-694

Davies: Chiropractic Pediatrics, a clinical handbook. Reprint 2000, Churchill Livingstone- Harcourt Publisher Limited, p.243-250, p. 268-269

Fysch: Chiropractic care for the pediatric patient. First Edition,2002. International Chiropractors Association on Chiropractic Pediatrics, p.259-267
