# Supplementary material for: Effectiveness of chiropractic manipulation versus sham manipulation on recurrent headaches in children aged 7–14 years, Protocol for a randomized clinical trial
Source: Chiropr Man Therap. 2019 Aug 23;27:40. doi: 10.1186/s12998-019-0262-y (PMC6706934; doi:10.1186/s12998-019-0262-y)
Supplement: Supplementary file 4 — a: Screening A. b: Screening B. (DOCX 24 kb) [file 12998_2019_262_MOESM4_ESM.docx]

Appendix 4

**Children with headache, aged 7-14**

**RCT SCREENING - 1. VISIT**

**ID-number:** _________ (according to NIKKB)

**Name:** __________________________________________

**Sex:** boy 🞎 girl 🞎

**Age:** ______

**Height:** ______ cm

**Weight:** ______ kg

**--------------------------------------------------------------------------------------------------------------------------------------------**

**1. Blood pressure:** _______ / _______

**2. Puls:** ________

**3. Fever:** yes 🞎 no 🞎

**4. Swollen lymph nodes, neck**: yes 🞎 no 🞎

**--------------------------------------------------------------------------------------------------------------------------------------------**

**5. Suspicion of abuse** (Physiological, psychological, medical) yes 🞎 no 🞎

Comments: _______________________________________________________________

**--------------------------------------------------------------------------------------------------------------------------------------------**

**6. Neurological examination** normal**:** yes 🞎 no 🞎

If no, describe: _______________________________________________________________

Reflexes of upper and lower extremities

Cranial nerves

Vibration test

Finger to nose test

Romberg sign

Walking on heels

Walking on toes

Walk on a straight line

Standing on one leg for 30 seconds

Squatting position

Hand pronation and supination

Heel to shin test

**7. Orthopedic examination,** normal**:** yes 🞎 no 🞎

If no, describe: _______________________________________________________________

**--------------------------------------------------------------------------------------------------------------------------------------------**

Cervical compression

Cervical distraction

Soto Hall

Passive ranges of motion in the cervical spine

Active ranges of motion in the cervical spine

**8. Red flags:** yes 🞎 no 🞎

If yes, describe: _______________________________________________________________

**--------------------------------------------------------------------------------------------------------------------------------------------**

Frequent and continuous vomiting_____

Acute vision problem_____

Acute stiffness of the neck (not biomechanical)_____

Weakness of the legs/momentary unconscious_____

Imbalance when walking____

Involuntary movements____

Extreme worsening of headache when standing up____

Inability to look upward_____

Papilloedema_____

Observed absence_____

If fever present:

Ability to bend one leg and extend it upwards in supine position (Kernigs sign)_____

Bending neck towards chest in supine position_____

**--------------------------------------------------------------------------------------------------------------------------------------------**

**9. Scoliosis:**

None 🞎 1

Functional scoliosis 🞎 2

Structural scoliosis 🞎 3

**--------------------------------------------------------------------------------------------------------------------------------------------**

**10. Muscle tone:**

Good 🞎 1

Hypo 🞎 2

Hyper 🞎 3

Asymmetrical 🞎 4

Comments: ____________________________________________________________________

__________________________________________________________________________________

**--------------------------------------------------------------------------------------------------------------------------------------------**

**11. Ranges of motion in the neck,** normal**:** yes 🞎 no 🞎 (if no, mark below)

|  |  | Symmetrical | Asymmetrical |
| --- | --- | --- | --- |
| Active ROM | Rotation |  |  |
|  | Lateral flexion |  |  |
| Passive ROM | Rotation |  |  |
|  | Lateral flexion |  |  |

**--------------------------------------------------------------------------------------------------------------------------------------------**

**--------------------------------------------------------------------------------------------------------------------------------------------**

**12. Chiropractic manual examination of spine,** normal**:** yes 🞎 no 🞎 (if dysfunction, mark below:)

|  | extension | flexion | right rot. | left rotation | right lat. flexion flex. | left lat. flex. |
| --- | --- | --- | --- | --- | --- | --- |
| Occiput |  |  |  |  |  |  |
| C1 |  |  |  |  |  |  |
| C2 |  |  |  |  |  |  |
| C3 |  |  |  |  |  |  |
| C4 |  |  |  |  |  |  |
| C5 |  |  |  |  |  |  |
| C6 |  |  |  |  |  |  |
| C7 |  |  |  |  |  |  |
| T1 |  |  |  |  |  |  |
| T2 |  |  |  |  |  |  |
| T3 |  |  |  |  |  |  |
| T4 |  |  |  |  |  |  |
| T5 |  |  |  |  |  |  |
| T6 |  |  |  |  |  |  |
| T7 |  |  |  |  |  |  |
| T8 |  |  |  |  |  |  |
| T9 |  |  |  |  |  |  |
| T10 |  |  |  |  |  |  |
| T11  T12 |  |  |  |  |  |  |
| T12 |  |  |  |  |  |  |
| L1 |  |  |  |  |  |  |
| L2 |  |  |  |  |  |  |
| L3 |  |  |  |  |  |  |
| L4 |  |  |  |  |  |  |
| L5 |  |  |  |  |  |  |
| S1 |  |  |  |  |  |  |
| S2 |  |  |  |  |  |  |
| S3 |  |  |  |  |  |  |
| S4 |  |  |  |  |  |  |
| Coccyx |  |  |  |  |  |  |

**--------------------------------------------------------------------------------------------------------------------------------------------**

**--------------------------------------------------------------------------------------------------------------------------------------------**

**13. Chiropractic manual examination of the sacroiliac articulations,** normal**:** yes 🞎 no 🞎 (if dysfunction, mark below:)

Decreased posterior flex. 🞎

Decreased anterior flex. 🞎

Decreased ext. rot. 🞎

Decreased int. rot. 🞎

**14. Chiropractic manual examination of the temporomandibular,** normal**:** yes 🞎 no 🞎 (if no, mark below)

Decreased right 🞎

Decreased left 🞎

**15. Chiropractic manual examination of the extremities,** normal**:** yes 🞎 no 🞎

If no, describe: _______________________________________________________________

**--------------------------------------------------------------------------------------------------------------------------------------------**

**16. Treatment will be given:**

| Occ. |  | C1 | C2 | C3 | C4 | C5 | C6 | C7 |
| --- | --- | --- | --- | --- | --- | --- | --- | --- |
|  |  |  |  |  |  |  |  |  |

| T1 | T2 | T3 | T4 | T5 | T6 | T7 | T8 | T9 | T10 | T11 | T12 |
| --- | --- | --- | --- | --- | --- | --- | --- | --- | --- | --- | --- |
|  |  |  |  |  |  |  |  |  |  |  |  |

| L1 | L2 | L3 | L4 | L5 |  | S1 | S2 | S3 | S4 |  | Coc. |
| --- | --- | --- | --- | --- | --- | --- | --- | --- | --- | --- | --- |
|  |  |  |  |  |  |  |  |  |  |  |  |

**17. Treatment will not be given (exclusion):**

Referred to pediatrician 🞎 1

Indication: __________________________________________________________________

No musculoskeletal dysfunction is identified 🞎 2

Comments: ________________________________________________________________

Other reason for exclusion 🞎 3

Describe: _______________________________________________________________________
