# Supplementary material for: Effectiveness of chiropractic manipulation versus sham manipulation on recurrent headaches in children aged 7–14 years, Protocol for a randomized clinical trial
Source: Chiropr Man Therap. 2019 Aug 23;27:40. doi: 10.1186/s12998-019-0262-y (PMC6706934; doi:10.1186/s12998-019-0262-y)
Supplement: Supplementary file 5 — Immediate referral to pediatrician (DOCX 13 kb) [file 12998_2019_262_MOESM5_ESM.docx]

Appendix 5

**Criteria for immediate referral to study pediatrician (exclusion)**

- Kinesthesia
- Pupillary dysfunction, bulging, papilledema
- Blood pressure above 120/80
- Suspicion of abuse (physical, psychological, medical)
- Underweight or suspicion of anorexia
- Continuous vomiting or vomiting without pain or nausea
- Loss of consciousness/black-outs/absence
- Significant increasing headache or nightly headache
