# Supplementary material for: Effectiveness of chiropractic manipulation versus sham manipulation on recurrent headaches in children aged 7–14 years, Protocol for a randomized clinical trial
Source: Chiropr Man Therap. 2019 Aug 23;27:40. doi: 10.1186/s12998-019-0262-y (PMC6706934; doi:10.1186/s12998-019-0262-y)
Supplement: Supplementary file 6 — Chiropractic examination (DOCX 12 kb) [file 12998_2019_262_MOESM6_ESM.docx]

Appendix 5

**Description of the chiropractic manual examination**

The chiropractic examination is in addition to the normal clinical examination of the child. It includes an analysis of the spine, pelvis and temporomandibular articulation and if relevant also of the extremities. During the examination the chiropractor will use manual static and motion palpation to determine if there is decreased mobility, stiffness and/or pain in one or more articulations when flexion, extension, lateral flexion and rotation is performed. During the examination each articulation is examined using digital pressure and compared to adjacent articulations. The intention is to identify articulations with significant decrease in mobility in one or more directions with or without pain. If one or more dysfunctional articulations are identified, they will receive spinal manipulation treatment in order to gradually reestablish as normal and pain free function as possible. Evaluation of posture, muscle tonus and atrophy will be included in the examination as well as evaluation of swelling and edema.
