# Supplementary material for: Effectiveness of chiropractic manipulation versus sham manipulation on recurrent headaches in children aged 7–14 years, Protocol for a randomized clinical trial
Source: Chiropr Man Therap. 2019 Aug 23;27:40. doi: 10.1186/s12998-019-0262-y (PMC6706934; doi:10.1186/s12998-019-0262-y)
Supplement: Supplementary file 7 — Advices given to all participants before randomization (DOCX 13 kb) [file 12998_2019_262_MOESM7_ESM.docx]

Appendix 7

**Advice given to all participants before randomization**

Dear participant

We want to encourage you to follow these guidelines during your four months participation in this trial:

1. Make sure to get at least 8 hours of sleep every night
2. Eat regularly: 3 meals per day and 3 small meals in between
3. Drink 2-3 liters of water per day, modified to size and physical activity
4. Make sure to exercise and get at least ½ hour of exercise per day
5. Limit your time in front of computer/I-pad/mobile phone/play station as much as possible and be aware of sitting up with a straight spine and neck when you use a screen.
6. Avoid the things you KNOW will give you a headache
7. If you are used to taking non-prescription medication for your headache, you are allowed to continue during this trial according to your needs.
