# Supplementary material for: Effectiveness of chiropractic manipulation versus sham manipulation on recurrent headaches in children aged 7–14 years, Protocol for a randomized clinical trial
Source: Chiropr Man Therap. 2019 Aug 23;27:40. doi: 10.1186/s12998-019-0262-y (PMC6706934; doi:10.1186/s12998-019-0262-y)
Supplement: Supplementary file 8 — Data collection at each visit on side effects and trauma (DOCX 14 kb) [file 12998_2019_262_MOESM8_ESM.docx]

Appendix 8

**Registration of side effects and trauma at each visit**

Reactions after last treatment:

|  | 0-2 hours | 2-24 hours | 24-48 hours | persistent |
| --- | --- | --- | --- | --- |
| tired |  |  |  |  |
| headache |  |  |  |  |
| soreness in treated area(s) |  |  |  |  |
| nausea |  |  |  |  |
| stomach pain |  |  |  |  |
| vomiting |  |  |  |  |
| visual or sensory disturbances (migraine) |  |  |  |  |
| visual or sensory disturbances upon arrival disappeared |  |  |  |  |
| headache upon arrival disappeared |  |  |  |  |
| more energy |  |  |  |  |
| feeling better |  |  |  |  |
| feeling better in treated area(s) |  |  |  |  |
| stomach pain upon arrival disappeared |  |  |  |  |
| other |  |  |  |  |

Trauma experienced since last visit: yes____ no____

|  | continued activity | stopped activity, rested, normal activity next day | absence from school/activities | Emergency room visit |
| --- | --- | --- | --- | --- |
| head |  |  |  |  |
| spine |  |  |  |  |
| extremity |  |  |  |  |
