# Supplementary material for: Effectiveness of chiropractic manipulation versus sham manipulation on recurrent headaches in children aged 7–14 years, Protocol for a randomized clinical trial
Source: Chiropr Man Therap. 2019 Aug 23;27:40. doi: 10.1186/s12998-019-0262-y (PMC6706934; doi:10.1186/s12998-019-0262-y)
Supplement: Supplementary file 9 — 1 year follow up questionnaire on headache (DOCX 29 kb) [file 12998_2019_262_MOESM9_ESM.docx]

Appendix 9

**Follow-up questionnaire, 1 year after entering the trial**

How often do you have a headache?

(4) ❑ Less than one day/month

(5) ❑ 1-3 days/month

(1) ❑ 1 - 2 days/week

(2) ❑ 3 - 5 days/week

(3) ❑ Almost every day

How long does your most typical headache last?

(1) ❑ Less than 2 hours

(2) ❑ From 2 hours to half a day

(3) ❑ All day

(4) ❑ All day and all night

When does your headache typically begin?

(1) ❑ Morning

(5) ❑ Before noon

(2) ❑ Afternoon

(3) ❑ Evening/night

(4) ❑ At different times

Where is your most common headache located?

(1) ❑ All over the head

(2) ❑ Backside of the head

(3) ❑ One or both sides of the head

(4) ❑ Forehead

(5) ❑ Behind one eye

(6) ❑ Different locations

(7) ❑ It varies a lot

Do you have other symptoms with your headache?

|  | Yes | No |
| --- | --- | --- |
| Nausea | (2) ❑ | (1) ❑ |
| Vomiting | (2) ❑ | (1) ❑ |
| Dizziness | (2) ❑ | (1) ❑ |
| Stomach pain | (2) ❑ | (1) ❑ |
| Visual disturbances | (2) ❑ | (1) ❑ |
| Spots in front of eyes | (2) ❑ | (1) ❑ |
| Tingling of arms or legs | (2) ❑ | (1) ❑ |
| Sensitive to light | (2) ❑ | (1) ❑ |
| Sensitive to sound | (2) ❑ | (1) ❑ |
| Others | (2) ❑ | (1) ❑ |

How often do you take non-prescription medication for headache (fx Panodil (paracetamol) or Ipren (NSAID))?

(5) ❑ Never

(2) ❑ 1 – 3 times/month

(3) ❑ 1 – 3 times/week

(4) ❑ More than 3 times/week

Do you take prescription medication for your headache?

(1) ❑ No

(2) ❑ Yes

What is the name of your prescription medication for headache?

________________________________________________________________________________

How often do you take it?

(2) ❑ 1 - 3 times/month

(3) ❑ 1 - 3 times/week

(4) ❑ More than 3 times/week

Do you take medication regularly for other conditions?

(2) ❑ Yes, (If yes, for what diseases?):_____

(1) ❑ No

On this scale 0= no pain and 10 is an extreme pain, that causes you to stop ongoing activity. Choose the number below that best describe your typical headache:

(1) ❑ 0=no pain

(2) ❑ 1

(3) ❑ 2

(4) ❑ 3

(5) ❑ 4

(6) ❑ 5

(7) ❑ 6

(8) ❑ 7

(9) ❑ 8

(10) ❑ 9

(11) ❑ 10=worst pain ever, stops your activity

After exiting the trial have you received any treatment for your headache?

(2) ❑ Yes

(1) ❑ No

Who has treated you?

|  | Yes | No |
| --- | --- | --- |
| Family doctor | (2) ❑ | (1) ❑ |
| Pediatrician | (2) ❑ | (1) ❑ |
| Physiotherapist | (2) ❑ | (1) ❑ |
| Chiropractor | (2) ❑ | (1) ❑ |
| Massage therapist | (2) ❑ | (1) ❑ |
| Reflexologist | (2) ❑ | (1) ❑ |
| Other | (2) ❑ | (1) ❑ |

How many times have you hurt your head and/or neck, causing you to contact family doctor or emergency room after exiting the trial?

(1) ❑ 0 times

(2) ❑ 1 - 3 times

(3) ❑ More than 3 times

How many times have you hurt your head and/or neck without consulting family doctor or emergency room after exiting the trial?

(1) ❑ 0 times

(2) ❑ 1 - 3 times

(3) ❑ More than 3 times

Have you been hospitalized due to head and/or neck trauma after exiting the trial?

(2) ❑ Yes

(1) ❑ No

Have you hurt your head and/or neck in one or more of these after exiting the trial?

|  | Yes | No |
| --- | --- | --- |
| Car accident | (1) ❑ | (2) ❑ |
| Fall off bike | (1) ❑ | (2) ❑ |
| Fall from more than 2 meters distance | (1) ❑ | (2) ❑ |
| Fall off/on trampoline | (1) ❑ | (2) ❑ |
| Fall off horse | (1) ❑ | (2) ❑ |
| Hit by another player in any contactsport | (1) ❑ | (2) ❑ |
| Experienced episode of violence to you | (1) ❑ | (2) ❑ |

Have you had any days off from school due to trauma after exiting the trial?

(3) ❑ Yes, more than once

(2) ❑ Yes, once

(1) ❑ No

Have you had a concussion after exiting the trial?

(1) ❑ Yes

(2) ❑ No

How many days off from school do you have on average?

(1) ❑ Less than 5 days/year

(2) ❑ 5 – 20 days/year

(3) ❑ more than 20/year

What are the most common reasons for your days off from school?

|  | Yes | No |
| --- | --- | --- |
| Headache | (1) ❑ | (2) ❑ |
| Neck pain | (1) ❑ | (2) ❑ |
| Back pain | (1) ❑ | (2) ❑ |
| Common cold | (1) ❑ | (2) ❑ |
| Earache | (1) ❑ | (2) ❑ |
| Stomach pain | (1) ❑ | (2) ❑ |
| Menstruation | (1) ❑ | (2) ❑ |
| Don’t want to go to school | (1) ❑ | (2) ❑ |
| \| Other reasons \| (1) ❑ \| (2) ❑ \| \| --- \| --- \| --- \| | (1) ❑ | (2) ❑ |

Other possible reasons for days off from school:

________________________________________
________________________________________
________________________________________
________________________________________
________________________________________
________________________________________

How many days off sick have you had the last year due to headache?

(1) ❑ 0 days

(2) ❑ 1 - 5 days

(3) ❑ 5 - 20 days

(4) ❑ More than 20 days

Who do you live with at your current adress?

(1) ❑ Mother and father

(2) ❑ Mother alone

(3) ❑ Father alone

(4) ❑ Mother and new partner

(5) ❑ Far and new partner

How many days during a 14 days period do you live at your current adress?

(1) ❑ All 14 days

(2) ❑ 10 - 13 days

(3) ❑ 7 - 9 days

(4) ❑ 5 - 6 days

(5) ❑ 1 - 4 days

What was the total income of this entire household before tax and deductions were withdrawn in 2015?

(1) ❑ Below kr. 200.000

(2) ❑ Kr. 200.000 - 299.999

(3) ❑ Kr. 300.000 - 399.999

(4) ❑ Kr. 400.000 - 499.999

(5) ❑ Kr. 500.000 - 599.999

(6) ❑ Kr. 600.000 - 699.999

(7) ❑ Kr. 700.000 - 799.999

(8) ❑ Kr. 800.000 or more

Thank you very much for your answers
